# Clinical and Microbiological Characteristics of Bacteremic Pneumonia Caused by *Klebsiella pneumoniae*

**DOI:** 10.3389/fcimb.2022.903682

**Published:** 2022-06-23

**Authors:** I-Ren Chen, Shih-Neng Lin, Xin-Ni Wu, Sheng-Hua Chou, Fu-Der Wang, Yi-Tsung Lin

**Affiliations:** ^1^ Division of Infectious Diseases, Department of Medicine, Taipei Veterans General Hospital, Taipei, Taiwan; ^2^ School of Medicine, National Yang Ming Chiao Tung University, Taipei, Taiwan; ^3^ Institute of Emergency and Critical Care Medicine, National Yang Ming Chiao Tung University, Taipei, Taiwan

**Keywords:** *Klebsiella pneumoniae*, community-acquired pneumonia (CAP), nosocomial pneumonia (NP), bacteremia, mortality

## Abstract

*Klebsiella pneumoniae* is a common pathogen of nosocomial pneumonia worldwide and community-acquired pneumonia (CAP) in Asia. Previous studies have shown that *K. pneumoniae* bacteremic CAP is associated with high mortality. We aimed to revisit *K. pneumoniae* bacteremic pneumonia in the current era and determine the risk factors associated with 28-day mortality. Between January 2014 and August 2020, adult patients with *K. pneumoniae* bacteremic pneumonia in a medical center in Taiwan were identified. Clinical and microbiological characteristics were compared between CAP and nosocomial pneumonia. Risk factors for 28-day mortality were analyzed using multivariate logistic regression. Among 150 patients with *K. pneumoniae* bacteremic pneumonia, 52 had CAP and 98 had nosocomial pneumonia. The 28-day mortality was 52% for all patients, 36.5% for CAP, and 60.2% for nosocomial pneumonia. Hypervirulent *K. pneumoniae* was more prevalent in CAP (61.5%) than in nosocomial pneumonia (16.3%). Carbapenem-resistant *K. pneumoniae* was more prevalent in nosocomial pneumonia (58.2%) than in CAP (5.8%). Nosocomial pneumonia, a higher Severe Organ Failure Assessment score, and not receiving appropriate definitive therapy were independent risk factors for 28-day mortality. In conclusion, revisiting *K. pneumoniae* bacteremic pneumonia in the current era showed a high mortality rate. Host factors, disease severity, and timely effective therapy affect the treatment outcomes of these patients.

## Introduction

*Klebsiella pneumoniae* is an important and common pathogen of hospital-acquired pneumonia (HAP) and ventilator-associated pneumonia (VAP) worldwide (Kalil et al., 2016). The emergence of multidrug-resistant (MDR) and carbapenem-resistant *K. pneumoniae* that cause nosocomial pneumonia represents a challenge for public health because of their limited treatment options (Rodríguez-Baño et al., 2018; Tamma et al., 2021). Notably, *K. pneumoniae* is also a common pathogen of community-acquired pneumonia (CAP) in Asian countries, while it is less common in other regions (Song et al., 2008; Metlay et al., 2019). A surveillance study conducted in eight Asian countries from 2002 to 2004 showed that *K. pneumoniae* accounted for 15.4% of CAP cases and was the second-leading cause of CAP after *Streptococcus pneumoniae* (29.2%) (Song et al., 2008).

Pneumonia caused by *K. pneumoniae* is usually associated with high mortality. In our previous study conducted from 2014 to 2016, the 28-day mortality rates of CAP and nosocomial pneumonia caused by *K. pneumoniae* were 27.9% and 36.9%, respectively (Juan et al., 2020). Bacteremia in patients with pneumonia is associated with a more critical disease course and higher mortality rate (Forstner et al., 2020). A study conducted in Japan from 2004 to 2012 showed that the 30-day mortality was 34.8% for patients with bacteremic pneumonia caused by *K. pneumoniae*, which is significantly higher than that of *K. pneumoniae*-associated non-bacteremic pneumonia (11.3%) (Ito et al., 2015). Our previous study conducted from 2001 to 2008 showed that *K. pneumoniae* surpasses *S. pneumoniae* as the dominant pathogen of bacteremic CAP in Taiwan, and the 28-day mortality is higher for *K. pneumoniae* (55.1%) than for *S. pneumoniae* (27.3%) infections (Lin et al., 2010). Another study in Taiwan similarly reported *K. pneumoniae* as the most common pathogen of bacteremic CAP in Taiwan from 2008 to 2013, accounting for 21.9% of all cases, with a high 30-day mortality of 54.1% (Yang et al., 2018). However, studies on bacteremic nosocomial pneumonia caused by *K. pneumoniae* are limited. Our previous study conducted in 2015 identified pneumonia as the source of bacteremia in 21.8% of patients with *K. pneumoniae* nosocomial bacteremia (Juan et al., 2019).

The high mortality in patients with bacteremic pneumonia caused by *K. pneumoniae* represents a major problem in Taiwan. The present study revisited the burden of *K. pneumoniae* bacteremic pneumonia in the current era. We aimed to investigate the clinical and microbiological characteristics of patients with bacteremic pneumonia and investigate the risk factors associated with 28-day mortality among these patients.

## Materials and Methods

### Study Population

This retrospective cohort study was conducted at the Taipei Veterans General Hospital, a tertiary care center in Taipei, Taiwan. Adult patients diagnosed with bacteremic pneumonia caused by *K. pneumoniae* between January 2014 and August 2020 were included. The diagnosis of pneumonia was based on the American Thoracic Society and the Infectious Diseases Society of America guidelines, which require new or progressive pulmonary infiltrates to be identified *via* chest radiography, and clinical symptoms of pulmonary infection, including new onset of fever, purulent sputum, leukocytosis, and decline in oxygenation (Kalil et al., 2016; Metlay et al., 2019). All patients who had at least one positive blood culture of *K. pneumoniae* drawn within 24 h of diagnosis of pneumonia were included. Patients with an infection focus other than pneumonia at the time of diagnosis were excluded. Patients with polymicrobial infections, defined as blood or respiratory cultures that contained pathogens other than *K. pneumoniae* within 48 h of diagnosis of pneumonia, were also excluded. For patients with multiple episodes of *K. pneumoniae* bacteremic pneumonia during the study period, only the first episode was included. The study was approved by the Institutional Review Board of Taipei Veterans General Hospital (2020-04-024BCF). Informed consent was waived due to the retrospective nature of the study.

CAP was defined as pneumonia that occurred before hospitalization or diagnosed within 48 h of hospitalization (Metlay et al., 2019). Nosocomial pneumonia included HAP (non-VAP) and VAP; HAP was defined as pneumonia occurring after at least 48 h of hospitalization, and VAP was defined as pneumonia occurring after at least 48 h of mechanical ventilation (Kalil et al., 2016). The guidelines by the Infectious Diseases Society of America have grouped HAP and VAP together when considering their treatment (Kalil et al., 2016), therefore we grouped HAP and VAP together as nosocomial pneumonia.

### Data Collection

Medical records of the patients were reviewed, and the following data were collected on the day of pneumonia diagnosis: age, sex, prior length of stay (LOS) for patients with nosocomial pneumonia, comorbidities, and prior antibiotic exposures within one month. An immunocompromised state was documented if the patient met one of the following criteria: neutropenia with an absolute neutrophil count <500/mm^3^ at the time of pneumonia diagnosis, steroid use with an equivalent dose of ≥10 mg prednisolone daily for more than one week within one month, chemotherapy within one month, or immunosuppressant use within one month. Malignancies that were actively treated with chemotherapy or under palliative treatment were documented.

Comorbidities were evaluated using the Charlson Comorbidity Index. Disease severity was evaluated within 24 h of pneumonia diagnosis using the Sequential Organ Failure Assessment (SOFA) score and the Acute Physiology and Chronic Health Evaluation II (APACHE II) score (Knaus et al., 1985; Vincent et al., 1996), and whether the patient exhibited septic shock.

Antimicrobial agents administered for at least 48 h after the diagnosis of pneumonia were recorded. The therapy was considered appropriate if at least one antibiotic with *in vitro* activity against the clinical isolate was administered at an appropriate dose, and adjusted for the patient’s renal function, if necessary. Appropriate empirical therapy was defined as appropriate therapy administered within 48 h of pneumonia diagnosis. Appropriate definitive therapy was defined as appropriate therapy administered within 24 h after antimicrobial susceptibility test results were obtained by the clinician (Juan et al., 2020). The primary interest of this study was the 28-day mortality.

### Microbiological Analysis

We used Matrix-assisted laser desorption-ionization time-of-flight mass spectrometry (bioMérieux, Marcy-l’Étoile, France) for the identification of *K. pneumoniae*. The minimal inhibitory concentration (MIC) of common antibiotics were evaluated by Vitek-2 (bioMérieux Inc., Durham, NC), and the results were interpreted according to the Clinical Laboratory and Standards Institute criteria (CLSI, 2022). The capsular genotypes and the presence of *rmpA*/*rmpA2* genes of the isolates were determined by polymerase chain reaction (PCR) as described previously (Pan et al., 2015; Russo et al., 2018). Hypervirulent *K. pneumoniae* strains were defined as strains with *rmpA* or *rmpA2* genes, as described previously (Russo et al., 2018; Russo and Marr, 2019). Antimicrobial-susceptible strains were defined as strains with non-susceptibility to ampicillin only (Juan et al., 2020). MDR *K. pneumoniae* strains were defined as strains with non-susceptibility to at least one agent in three or more antimicrobial categories (Magiorakos et al., 2012). Plasmid-borne extended spectrum beta-lactamase (ESBL) genes (encoding CTX-M, TEM, and SHV) and AmpC-like genes (encoding CMY and DHA) were detected by PCR, as described previously (Lin et al., 2014). For carbapenem-resistant strains, the presence of carbapenemase genes were detected by PCR (encoding Ambler class A families KPC, Ambler class B families NDM, VIM, and IMP, and Ambler class D family OXA-48-type) as described previously (Lin et al., 2019).

### Statistical Analysis

Categorical variables were evaluated using the Fisher’s exact test. Continuous variables were evaluated using the Mann–Whitney U test. To evaluate the risk factors associated with 28-day mortality, variables with a value of *P* < 0.1 in univariate analysis, after checking for collinearity, were selected *via* backward elimination in a multivariate logistic regression model. The fitness of the model was tested using the Hosmer–Lemeshow goodness-of-fit test. A two-tailed *P* < 0.05 value was considered significant. All analyses were performed using R software (v3.6.2, R core team, Vienna, Austria).

## Results

From January 2014 to August 2020, 182 patients with *K. pneumoniae* bacteremic pneumonia were identified, among which 32 were excluded because of polymicrobial infections, and 150 patients were finally enrolled. Among the 150 patients, 52 (34.7%) had CAP and 98 (65.3%) had nosocomial pneumonia (including 48 with HAP, and 50 with VAP). The patients had a median age of 77.5 (interquartile range [IQR], 63–85.8); 114 patients (76%) were men. The median Charlson Comorbidity Index was 8 (IQR, 6.2–10). The patients presented with high disease severity, as the median SOFA score was 10 (IQR, 6–13), the median APACHE II score was 30 (IQR, 23–35), and 97 patients (64.7%) presented with septic shock within 24 h of pneumonia diagnosis. The 28-day mortality was 52% for all patients, 36.5% for CAP, and 60.2% for nosocomial pneumonia. The clinical characteristics of the patients are summarized in **Table 1**. Univariate analyses showed that compared to patients with CAP, patients with nosocomial pneumonia were younger, more frequently immunocompromised, had more prior exposure to antibiotics, were less frequently treated with appropriate empirical and definitive therapy, and had higher 28-day mortality.

**Table 1:** Clinical characteristics of patients with *K. pneumoniae* bacteremic pneumonia.

Variable	All patients n=150	CAP n=52 (34.7%)	Nosocomial pneumonia n=98 (65.3%)	*P* value
**Demographics**				
Age	77.5 (63-85.8)	80.5 (69.2-86.2)	73 (60.2-85)	**0.025**
Male sex	114 (76)	41 (78.8)	73 (74.5)	0.688
**Underlying disease**				
Charlson Comorbidity Index	8 (6.2-10)	8 (6.8-9.2)	9 (6.2-11)	0.366
Diabetes mellitus	55 (36.7)	20 (38.5)	35 (35.7)	0.859
Congestive heart failure	31 (20.7)	10 (19.2)	21 (21.4)	0.834
Chronic kidney disease	47 (31.3)	17 (32.7)	30 (30.6)	0.854
Hemodialysis	24 (16)	4 (7.7)	20 (20.4)	0.060
Liver cirrhosis	11 (7.3)	4 (7.7)	7 (7.1)	1.000
Cerebral vascular disease	40 (26.7)	16 (30.8)	24 (24.5)	0.441
Malignancy	68 (45.3)	21 (40.4)	47 (48)	0.394
Immunosuppression	60 (40)	13 (25)	47 (48)	**0.008**
**Prior antibiotic exposure**				
Any antibiotic	87 (58)	12 (23.1)	75 (76.5)	**<0.001**
1st/2nd generation CEF	13 (8.7)	4 (7.7)	9 (9.2)	1.000
3rd/4th generation CEF	40 (26.7)	6 (11.5)	34 (34.7)	**0.002**
BLBLI	48 (32)	7 (13.5)	41 (41.8)	**<0.001**
Carbapenem	46 (30.7)	1 (1.9)	45 (45.9)	**<0.001**
Fluoroquinolone	36 (24)	5 (9.6)	31 (31.6)	**0.002**
Aminoglycoside	10 (6.7)	1 (1.9)	9 (9.2)	0.166
**Disease severity**				
SOFA score	10 (6-13)	10.5 (6-13)	10 (6-14)	0.673
APACHE II score	30 (23-35)	30 (23.8-35)	29.5 (23-35.8)	0.756
Septic shock at diagnosis	97 (64.7)	35 (67.3)	62 (63.3)	0.720
**Treatment**				
Appropriate empirical therapy	123 (82)	48 (92.3)	75 (76.5)	**0.024**
Appropriate definite therapy	135 (90)	51 (98.1)	84 (85.7)	**0.020**
**Outcome**				
28-day mortality	78 (52)	19 (36.5)	59 (60.2)	**0.006**

Data are presented as median (interquartile range) for continuous variables, and number (percent) for categorical variables. Categorical variables were evaluated using the Fisher’s exact test. Continuous variables were evaluated using the Mann–Whitney U test. Statistically significant P values are highlighted in bold. CAP, community-acquired pneumonia; CEF, cephalosporin; BLBLI, β-lactam-β-lactamase inhibitor.

The antimicrobial susceptibility and microbiological characteristics of the 150 K*. pneumoniae* isolates are presented in **Table 2**. Except for amikacin, the susceptibility rates for all antibiotics were higher for CAP isolates than those for nosocomial pneumonia isolates. Antimicrobial-susceptible *K. pneumoniae* was more frequently isolated from CAP (78.8%; 95% confidence interval [CI], 67.7%–89.9%) than from nosocomial pneumonia (24.5%; 95% CI, 16.0%–33.0%). Among all *K. pneumoniae* isolates, K2 (10.7%) was the most common capsular type, followed by K1 (9.3%). Capsular types K1, K2, K5, K20, K54, and K57 were more commonly found in CAP isolates (53.8%; 95% CI, 40.3%–67.4%) than in nosocomial pneumonia isolates (15.3%; 95% CI, 8.2%–22.4%). A total of 48 hypervirulent strains were identified among the *K. pneumoniae* isolates. All 30 strains with capsular types K1/K2 and 10/13 (76.9%) strains with capsular types K5, K20, K54, and K57 were identified as hypervirulent strains. Among the hypervirulent strains, 43 (89.6%) were antimicrobial-susceptible strains, and 3 (6.2%) were MDR strains. Hypervirulent strains were more commonly found in CAP (61.5%; 95% CI, 48.3%–74.8%) than in nosocomial pneumonia (16.3%; 95% CI, 9.0%–23.6%). MDR *K. pneumoniae* was more frequently isolated from nosocomial pneumonia (71.4%; 95% CI, 62.5%–80.4%) than from CAP (19.2%; 95% CI, 8.5%–29.9%). Carbapenem-resistant *K. pneumoniae* was also more frequently isolated from nosocomial pneumonia (58.2%; 95% CI, 48.4%–67.9%) than from CAP (5.8%; 95% CI, 0.0%–12.1%). As for resistance mechanisms, ESBL and AmpC beta-lactamase-producing strains were more commonly found in nosocomial pneumonia (26.5%; 95% CI, 17.8%–35.3%) than in CAP (7.7%; 95% CI, 0.4%–14.9%). Among the 98 isolates obtained from patients with nosocomial pneumonia, 47 (48%) produced carbapenemases, among which *K. pneumoniae* carbapenemase-2 was the predominant carbapenemase (43/47, 91.5%). Carbapenemase-producing *K. pneumoniae* was not isolated from CAP.

**Table 2:** Antimicrobial susceptibility and microbiological characteristics of the *K. pneumoniae* clinical isolates.

Variable	All patients n=150	CAP n=52 (34.7%)	Nosocomial pneumonia n=98 (65.3%)	*P* value
**Antimicrobial susceptibility**				
Cefuroxime	70 (46.7)	43 (82.7)	27 (27.6)	**<0.001**
Ceftazidime	73 (48.7)	46 (88.5)	27 (27.6)	**<0.001**
Cefepime	86 (57.3)	51 (98.1)	35 (35.7)	**<0.001**
Piperacillin/Tazobactam	77 (51.3)	47 (90.4)	30 (30.6)	**<0.001**
Ciprofloxacin	72 (48)	44 (84.6)	28 (28.6)	**<0.001**
Levofloxacin	68 (45.3)	41 (78.8)	27 (27.6)	**<0.001**
Ertapenem	91 (60.7)	50 (96.2)	41 (41.8)	**<0.001**
Imipenem	99 (66)	52 (100)	47 (48)	**<0.001**
Amikacin	144 (96)	51 (98.1)	93 (94.9)	0.665
Gentamicin	111 (74)	48 (92.3)	63 (64.3)	**<0.001**
**Capsular types**				
K1	14 (9.3)	10 (19.2)	4 (4.1)	**0.006**
K2	16 (10.7)	10 (19.2)	6 (6.1)	**0.023**
K1 and K2	30 (20)	20 (38.5)	10 (10.2)	**<0.001**
K1, K2, K5, K20, K54, and K57	43 (28.7)	28 (53.8)	15 (15.3)	**<0.001**
Hypervirulent *K. pneumoniae*	48 (32)	32 (61.5)	16 (16.3)	**<0.001**
Antimicrobial susceptible *K. pneumoniae*	65 (43.3)	41 (78.8)	24 (24.5)	**<0.001**
MDR *K. pneumoniae*	80 (53.3)	10 (19.2)	70 (71.4)	**<0.001**
Carbapenem resistant *K. pneumoniae*	60 (40)	3 (5.8)	57 (58.2)	**<0.001**
**Resistance mechanisms**				
ESBL/AmpC-producing strains	30 (20)	4 (7.7)	26 (26.5)	**0.005**
Carbapenemase-producing strains	47 (31.3)	0 (0)	47 (48)	**-**
KPC-2-producing strains	43 (28.7)	0 (0)	43 (43.9)	**-**
OXA-48-producing strains	4 (2.7)	0 (0)	4 (4.1)	**-**

Data are presented as number (percent), and evaluated using the Fisher’s exact test. Statistically significant P values are highlighted in bold. CAP, community-acquired pneumonia; MDR, multidrug-resistant; ESBL, extended-spectrum beta-lactamase; KPC, Klebsiella pneumoniae carbapenemase.

The clinical and microbiological characteristics of patients with nosocomial *K. pneumoniae* bacteremic pneumonia are presented in **Table 3**. Univariate analyses showed that compared to patients with HAP, those with VAP had longer length of stay before pneumonia, lower Charlson Comorbidity Index, were more frequently under hemodialysis, less frequently diagnosed with malignancy, and had more prior exposures to antibiotics, especially carbapenems. Hypervirulent *K. pneumoniae* was more frequently isolated from patients with HAP than those with VAP, while ESBL/AmpC-producing strains and Carbapenemase-producing strains were more frequently isolated from patients with VAP than those with HAP. No significant difference in 28-day mortality was observed between HAP and VAP cases.

**Table 3:** Characteristics of patients with nosocomial bacteremic pneumonia.

Variable	HAP (non-VAP) n=48 (49%)	VAP n=50 (51%)	P value
**Demographics**			
Age	76.5 (62.8-85)	69.5 (56.2-85)	0.363
Male sex	36 (75)	37 (74)	1.000
LOS before pneumonia, days	15 (7-31.5)	31 (11.5-94.8)	**0.010**
**Underlying disease**			
Charlson Comorbidity Index	9 (7-11)	8 (5-10)	**0.044**
Diabetes mellitus	21 (43.8)	14 (28)	0.140
Congestive heart failure	9 (18.8)	12 (24)	0.625
Chronic kidney disease	14 (29.2)	16 (32)	0.828
Hemodialysis	5 (10.4)	15 (30)	**0.023**
Liver cirrhosis	2 (4.2)	5 (10)	0.436
Cerebral vascular disease	11 (22.9)	13 (26)	0.816
Malignancy	29 (60.4)	18 (36)	**0.026**
Immunosuppression	19 (39.6)	28 (56)	0.112
**Prior antibiotic exposure**			
Any antibiotic	31 (64.6)	44 (88)	**0.008**
1st/2nd generation CEF	4 (8.3)	5 (10)	1.000
3rd/4th generation CEF	12 (25)	22 (44)	0.058
BLBLI	19 (39.6)	22 (44)	0.687
Carbapenem	14 (29.2)	31 (62)	**0.001**
Fluoroquinolone	11 (22.9)	20 (40)	0.084
Aminoglycoside	3 (6.2)	6 (12)	0.487
**Disease severity**			
SOFA score	9 (6.8-12)	11 (6-15.8)	0.080
APACHE II score	27.5 (19.8-37)	31.5 (24.2-35)	0.141
Septic shock at diagnosis	29 (60.4)	33 (66)	0.676
**Microbiological characteristics**			
Hvpervirulent KP	13 (27.1)	3 (6)	**0.006**
ESBL/AmpC-producing KP	8 (16.7)	18 (36)	**0.040**
Carbapenemase-producing KP	16 (33.3)	31 (62)	**0.005**
**Treatment**			
Appropriate empirical therapy	34 (70.8)	41 (82)	0.237
Appropriate definite therapy	39 (81.2)	45 (90)	0.257
**Outcome**			
28-day mortality	33 (68.8)	26 (52)	0.103

Data are presented as median (interquartile range) for continuous variables, and number (percent) for categorical variables. Categorical variables were evaluated using the Fisher’s exact test. Continuous variables were evaluated using the Mann–Whitney U test. Statistically significant P values are highlighted in bold. HAP, hospital-acquired pneumonia; VAP, ventilator-associated pneumonia; LOS, length of stay; CEF, cephalosporin; BLBLI, β-lactam-β-lactamase inhibitor; KP, Klebsiella pneumoniae; ESBL, extended-spectrum beta-lactamase.

The clinical characteristics of the 28-day survivors and non-survivors are presented in **Table 4**. Univariate analyses showed that nosocomial pneumonia, Charlson Comorbidity Index, prior exposure to antibiotics (3rd or 4th generation cephalosporins, beta-lactam-beta-lactamase inhibitors, and carbapenems), SOFA score, APACHE II score, septic shock, and carbapenemase-producing *K. pneumoniae* were associated with a higher 28-day mortality; hypervirulent *K. pneumoniae* and appropriate definitive therapy were associated with a lower 28-day mortality. In multivariate analysis, nosocomial pneumonia (adjusted odds ratio [aOR], 2.5; 95% CI, 1.09–5.75; *P* = 0.031) and SOFA score (aOR, 1.29; 98% CI, 1.16–1.44; *P* < 0.001) were positive predictors of 28-day mortality, and appropriate definitive therapy (aOR, 0.08; 95% CI, 0.01–0.75; *P* = 0.026) was a protective factor against 28-day mortality (**Table 5**).

**Table 4:** Characteristics of 28-day survivors and non-survivors.

Variable	Survivors n=72 (48%)	Non-survivors n=78 (52%)	P value
**Demographics**			
Age	75 (63-83.5)	80 (62.2-87)	0.100
Male sex	58 (80.6)	56 (71.8)	0.253
Nosocomial pneumonia	39 (54.2)	59 (75.6)	**0.006**
**Underlying disease**			
Charlson Comorbidity Index	8 (5-9.2)	9 (7-11)	**0.001**
Diabetes mellitus	28 (38.9)	27 (34.6)	0.614
Congestive heart failure	14 (19.4)	17 (21.8)	0.841
Chronic kidney disease	20 (27.8)	27 (34.6)	0.384
Hemodialysis	9 (12.5)	15 (19.2)	0.276
Liver cirrhosis	5 (6.9)	6 (7.7)	1.000
Cerebral vascular disease	18 (25)	22 (28.2)	0.714
Malignancy	27 (37.5)	41 (52.6)	0.073
Immunosuppression	23 (31.9)	37 (47.4)	0.067
**Prior antibiotic exposure**			
Any antibiotic	36 (50)	51 (65.4)	0.069
1st/2nd generation CEF	9 (12.5)	4 (5.1)	0.148
3rd/4th generation CEF	11 (15.3)	29 (37.2)	**0.003**
BLBLI	17 (23.6)	31 (39.7)	**0.037**
Carbapenem	15 (20.8)	31 (39.7)	**0.014**
Fluoroquinolone	17 (23.6)	19 (24.4)	1.000
Aminoglycoside	5 (6.9)	5 (6.4)	1.000
**Disease severity**			
SOFA score	7 (5-11)	12 (9-15.8)	**<0.001**
APACHE II score	25 (19.8-31.2)	33 (27.2-38)	**<0.001**
Septic shock at diagnosis	34 (47.2)	63 (80.8)	**<0.001**
**Microbiological characteristics**			
Hvpervirulent KP	30 (41.7)	18 (23.1)	**0.022**
ESBL/AmpC-producing KP	10 (13.9)	20 (25.6)	0.101
Carbapenemase-producing KP	16 (22.2)	31 (39.7)	**0.023**
**Treatment**			
Appropriate empirical therapy	61 (84.7)	62 (79.5)	0.524
Appropriate definite therapy	71 (98.6)	64 (82.1)	**0.001**

Data are presented as median (interquartile range) for continuous variables, and number (percent) for categorical variables. Categorical variables were evaluated using the Fisher’s exact test. Continuous variables were evaluated using the Mann–Whitney U test. Statistically significant P values are highlighted in bold. CEF, cephalosporin; BLBLI, β-lactam-β-lactamase inhibitor; KP, Klebsiella pneumoniae; ESBL, extended-spectrum beta-lactamase.

**Table 5:** Logistic regressions for variables associated with 28-day mortality.

Variable	Univariate		Multivariate	
	*P* value	OR (95% CI)	*P* value	aOR (95% CI)
Nosocomial pneumonia	**0.006**	2.63 (1.31-5.26)	**0.031**	2.5 (1.09-5.75)
Charlson Comorbidity Index	**0.001**	1.23 (1.09-1.38)	0.071	1.14 (0.99-1.32)
Malignancy	0.065	1.85 (0.96-3.54)	0.080	2.25 (0.91-5.56)
Immunosuppression	0.054	1.92 (0.99-3.74)	–	–
Previous exposure, 3^rd^/4^th^ CEF	**0.003**	3.28 (1.49-7.23)	–	–
Previous exposure, BLBLI	**0.036**	2.13 (1.05-4.33)	–	–
Previous exposure, carbapenem	**0.013**	2.51 (1.21-5.19)	–	–
SOFA score	**<0.001**	1.25 (1.14-1.37)	**<0.001**	1.29 (1.16-1.44)
APACHE II score	**<0.001**	1.11 (1.06-1.16)	–	–
Septic shock at diagnosis	**<0.001**	4.69 (2.26-9.73)	–	–
Hvpervirulent KP	**0.016**	0.42 (0.21-0.85)	–	–
Carbapenemase-producing KP	**0.022**	2.31 (1.13-4.73)	–	–
Appropriate definitive therapy	**0.009**	0.06 (0.01-0.5)	**0.026**	0.08 (0.01-0.75)

Statistically significant P values are highlighted in bold. OR, odds ratio; CI confidence interval; aOR, adjusted odds ratio; CEF, cephalosporin; BLBLI, β-lactam-β-lactamase inhibitor; KP, Klebsiella pneumoniae.

For patients with CAP, univariate analyses showed that the SOFA score, APACHE II score, and septic shock were associated with a higher 28-day mortality (Additional file 1: **Table S1**). In multivariate analysis, the APACHE II score (aOR, 1.1; 95% CI, 1.01-1.21; *P*=0.037) represented a risk factor for 28-day mortality (Additional file 1: **Table S2**).

For patients with nosocomial pneumonia, univariate analyses showed that the Charlson Comorbidity Index, prior exposures to 3rd or 4th generation cephalosporins, SOFA score, APACHE II score, and septic shock were associated with a higher 28-day mortality; appropriate definitive therapy was associated with a lower 28-day mortality (Additional file 1: **Table S3**). In multivariate analysis, the Charlson Comorbidity Index (aOR, 1.28; 95% CI, 1.08-1.51; *P*=0.004) and SOFA score (aOR, 1.32; 95% CI, 1.15-1.52; *P*<0.001) represented risk factors for 28-day mortality; appropriate definitive therapy (aOR, 0.1; 95% CI, 0.01-0.96; *P*=0.046) was a protective factor against 28-day mortality (Additional file 1: **Table S4**).

## Discussion

In this study, we revisited the clinical and microbiological characteristics of *K. pneumoniae* bacteremic pneumonia in Taiwan in recent years. We found a remarkably high 28-day mortality in the patients, especially for nosocomial pneumonia. Host factors and disease severity were found to markedly affect the treatment outcomes of patients with *K. pneumoniae* bacteremic pneumonia. Additionally, timely and effective therapy is essential to improve the survival of these patients.

*K. pneumoniae* is a common pathogen of CAP in Asian countries. We previously showed that the 28-day mortality of bacteremic CAP caused by *K. pneumoniae* was 55.1% between 2001 and 2008 in our hospital, and capsular type K1/K2 comprised half of the clinical isolates (Lin et al., 2010). We revisited the burden of pneumonia caused by *K. pneumoniae* from 2014 to 2020 in the present study and found that the 28-day mortality of bacteremic CAP caused by *K. pneumoniae* was 36.5%, and capsular type K1/K2 comprised 38.5% of the clinical isolates. Recent advances in clinical care may be responsible for the decreased mortality rates; however, these findings highlight the serious health issues of *K. pneumoniae* infections in Taiwan. Capsular types K1/K2 remained prevalent among the *K. pneumoniae* isolates that caused bacteremic CAP, consistent with the endemic nature of these strains in Taiwan (Lin et al., 2010). The presence of such capsular types of *K. pneumoniae* in the nasopharynx (Lin et al., 2015), and the possibility of community spreading continue to represent challenging issues. Moreover, it is alarming that MDR *K. pneumoniae* comprised 19.2% of the strains isolated from bacteremic CAP in the current study, while no MDR *K. pneumoniae* strains were isolated from bacteremic CAP in the previous study from 2001 to 2008 (Lin et al., 2010). One recent study in Taiwan also showed that bacteremia-causing *K. pneumoniae* strains isolated in 2017 had increased resistance to most classes of antimicrobial agents compared to those isolated in 2007 (Huang et al., 2020). Notably, in our study, 23.1% of the patients with bacteremic CAP were exposed to antibiotics within a month before the CAP episode. Antimicrobial exposure may partially account for the increased antimicrobial resistance in the current era, and highlights the urgent need to manage antimicrobial resistance in communities.

In our previous study from 2014 to 2016, we demonstrated that hypervirulent strains comprised 79.4% of the *K. pneumoniae* isolates that caused CAP and 41% of the *K. pneumoniae* isolates that caused nosocomial pneumonia (Juan et al., 2020). In the current study, hypervirulent strains comprised 61.5% and 16.3% of the *K. pneumoniae* isolates that caused bacteremic CAP and nosocomial pneumonia, respectively. MDR strains comprised 6.2% of the hypervirulent strains in the current study, similar to our previous findings from 2014 to 2016 (5.1%) (Juan et al., 2020). Our findings suggest that hypervirulent *K. pneumoniae* strains remain common in both community and hospital settings in Taiwan.

In our previous study conducted from 2014 to 2016 on pneumonia caused by *K. pneumoniae*, MDR *K. pneumoniae* comprised 49.3% of the clinical isolates of nosocomial pneumonia, and carbapenem-resistant *K. pneumoniae* comprised 24.6% of the isolates (Juan et al., 2020). In our current study, the proportion of MDR *K. pneumoniae* and carbapenem-resistant *K. pneumoniae* increased to 71.4% and 58.2%, respectively. Therefore, the antimicrobial resistance of *K. pneumoniae* in nosocomial pneumonia during the past five years in Taiwan has increased to an alarming extent. Nosocomial pneumonia itself is an independent risk factor for 28-day mortality in all cases, and the increased spread of antimicrobial-resistant strains represents a challenging issue for physicians. When comparing the characteristics of CAP and nosocomial pneumonia, MDR and carbapenem-resistant *K. pneumoniae* were more commonly isolated in nosocomial pneumonia than in CAP. It may be associated with more common previous exposures to antibiotics among patients with nosocomial pneumonia compared to those with CAP.

The mortality associated with *K. pneumoniae* bacteremic nosocomial pneumonia has not been previously reported, and we demonstrated for the first time a high 28-day mortality of 60.2% among these patients, despite the advanced clinical care in the current era. Notably, appropriate definitive therapy was a protective factor against 28-day mortality for all patients and for those with nosocomial pneumonia, emphasizing the importance of timely effective therapy according to the microbial culture and antimicrobial sensitivity results. A recent study evaluating bacteremia caused by carbapenemase-producing *Enterobacterales*, among which 86% were *K. pneumoniae*, also showed that appropriate definitive therapy is a protective factor against 30-day mortality (Gutiérrez-Gutiérrez et al., 2017). In addition, a higher SOFA score was also a risk factor of 28-day mortality. However, the microbiological characteristics of the clinical isolates, such as hypervirulence or carbapenem resistance, were not significantly associated with mortality. This indicates that host factors, disease severity, and timely appropriate therapy play more important roles in determining the outcomes of these patients.

Our study has a few limitations. First, this single-center study design may represent the epidemiology in Taiwan alone; however, the findings are valuable given the global increase in carbapenem-resistant *K. pneumoniae* prevalence and the high prevalence of hypervirulent *K. pneumoniae* in Asia. Second, the retrospective study design may contain selection bias. For example, patients with severe pneumonia and undiagnosed bacteremia might not have been included. Moreover, the relatively old age of our study population, and the initial reasons of hospitalization for patients with nosocomial pneumonia may have affected the mortality rate. Third, we only used *rmpA*/*rmpA2* genes to identify hypervirulent *K. pneumoniae*, and the consensus of the markers for hypervirulent *K. pneumoniae* was not concluded in the literature (Zhu et al., 2021). Fourth, more than half of the nosocomial isolates were resistant to carbapenem, and novel antimicrobial agents against carbapenem-resistant *K. pneumoniae*, such as ceftazidime-avibactam, were not available during the study period (Shields et al., 2017; Yahav et al., 2020). Further studies are necessary to identify the benefits of novel agents in the treatment of bacteremic nosocomial pneumonia caused by carbapenem-resistant *K. pneumoniae*. However, our findings are valuable since these new agents remain unavailable in many countries.

In conclusion, revisiting *K. pneumoniae* bacteremic pneumonia in Taiwan in the current era showed a high mortality rate, especially for nosocomial pneumonia. Nosocomial pneumonia, SOFA score, and lack of appropriate definitive treatment were positive predictors of 28-day mortality among patients with *K. pneumoniae* bacteremic pneumonia. The increased proportion of carbapenem-resistant *K. pneumoniae* among strains that cause bacteremic nosocomial pneumonia is a serious problem, and studies on novel antibiotics against carbapenem-resistant *K. pneumoniae* are necessary.

## Data Availability Statement

The original contributions presented in the study are included in the article/**Supplementary Material**. Further inquiries can be directed to the corresponding author.

## Ethics Statement

The studies involving human participants were reviewed and approved by the Institutional Review Board of Taipei Veterans General Hospital. Written informed consent for participation was not required for this study in accordance with the national legislation and the institutional requirements.

## Author Contributions

Y-TL and F-DW designed and supervised the study. I-RC, S-NL, and X-NW participated in the data collection. Y-TL and S-HC participated in the laboratory experiment. I-RC and Y-TL analyzed the data and drafted the manuscript. All authors read and approved the final manuscript.

## Funding

This work was supported by grants from the Ministry of Science and Technology in Taiwan (MOST 108-2314-B-010-030-MY3); Taipei Veterans General Hospital (V111A-010, V109E-004-2, V110E-004-2, V110C-068, and V111C-061).

## Conflict of Interest

The authors declare that the research was conducted in the absence of any commercial or financial relationships that could be construed as a potential conflict of interest.

## Publisher’s Note

All claims expressed in this article are solely those of the authors and do not necessarily represent those of their affiliated organizations, or those of the publisher, the editors and the reviewers. Any product that may be evaluated in this article, or claim that may be made by its manufacturer, is not guaranteed or endorsed by the publisher.

## References

[B1] CLSI (2022). Performance Standards for Antimicrobial Susceptibility Testing. 32nd ed (CLSI Supplement M100). (Wayne, PA: Clinical and Laboratory Standards Institute).

[B2] Forstner C. Patchev V. Rohde G. Rupp J. Witzenrath M. Welte T.. (2020). Rate and Predictors of Bacteremia in Afebrile Community-Acquired Pneumonia. Chest. 157 (3), 529–539. doi: 10.1016/j.chest.2019.10.006 31669433

[B3] Gutiérrez-Gutiérrez B. Salamanca E. de Cueto M. Hsueh P. R. Viale P. Paño-Pardo J. R.. (2017). Effect of Appropriate Combination Therapy on Mortality of Patients With Bloodstream Infections Due to Carbapenemase-Producing *Enterobacteriaceae* (INCREMENT): A Retrospective Cohort Study. Lancet Infect. Dis. 17 (7), 726–734. doi: 10.1016/S1473-3099(17)30228-1 28442293

[B4] Huang Y. T. Chen C. S. Chen H. A. Hsu H. S. Liang M. H. Chang M. H.. (2020). *Klebsiella Pneumoniae* Bacteremia Revisited: Comparison Between 2007 and 2017 Prospective Cohorts at a Medical Center in Taiwan. J. Infect. 81 (5), 753–757. doi: 10.1016/j.jinf.2020.08.039 32860818

[B5] Ito R. Shindo Y. Kobayashi D. Ando M. Jin W. Wachino J.. (2015). Molecular Epidemiological Characteristics of *Klebsiella Pneumoniae* Associated With Bacteremia Among Patients With Pneumonia. J. Clin. Microbiol. 53 (3), 879–886. doi: 10.1128/JCM.03067-14 25568434 PMC4390620

[B6] Juan C. H. Chuang C. Chen C. H. Li L. Lin Y. T. (2019). Clinical Characteristics, Antimicrobial Resistance and Capsular Types of Community-Acquired, Healthcare-Associated, and Nosocomial *Klebsiella Pneumoniae* Bacteremia. Antimicrob. Resist. Infect. Control. 8, 1. doi: 10.1186/s13756-018-0426-x 30622702 PMC6318907

[B7] Juan C. H. Fang S. Y. Chou C. H. Tsai T. Y. Lin Y. T. (2020). Clinical Characteristics of Patients With Pneumonia Caused by *Klebsiella Pneumoniae* in Taiwan and Prevalence of Antimicrobial-Resistant and Hypervirulent Strains: A Retrospective Study. Antimicrob. Resist. Infect. Control. 3, 9(1):4. doi: 10.1186/s13756-019-0660-x PMC6942382 31911832

[B8] Kalil A. C. Metersky M. L. Klompas M. Muscedere J. Sweeney D. A. Palmer L. B.. (2016). Management of Adults With Hospital-Acquired and Ventilator-Associated Pneumonia: 2016 Clinical Practice Guidelines by the Infectious Diseases Society of America and the American Thoracic Society. Clin. Infect. Dis. 63 (5), e61–e111. doi: 10.1093/cid/ciw353 27418577 PMC4981759

[B9] Knaus W. A. Draper E. A. Wagner D. P. Zimmerman J. E. (1985). APACHE II: A Severity of Disease Classification System. Crit. Care Med. 10), 818–829. doi: 10.1097/00003246-198510000-00009 3928249

[B10] Lin Y. T. Jeng Y. Y. Chen T. L. Fung C. P. (2010). Bacteremic Community-Acquired Pneumonia Due to *Klebsiella Pneumoniae*: Clinical and Microbiological Characteristics in Taiwan 2001-2008. BMC Infect. Dis. 10, 307. doi: 10.1186/1471-2334-10-307 20973971 PMC2987304

[B11] Lin Y. T. Su C. F. Chuang C. Lin J. C. Lu P. L. Huang C. T.. (2019). Appropriate Treatment for Bloodstream Infections Due to Carbapenem-Resistant *Klebsiella Pneumoniae* and *Escherichia Coli*: A Nationwide Multicenter Study in Taiwan. Open Forum Infect. Dis. 6, ofy336. doi: 10.1093/ofid/ofy336 30740468 PMC6362312

[B12] Lin Y. T. Wang F. D. Chan Y. J. Fu Y. C. Fung C. P. (2014). Clinical and Microbiological Characteristics of Tigecycline non-Susceptible *Klebsiella Pneumoniae* Bacteremia in Taiwan. BMC Infect. Dis. 14, 1. doi: 10.1186/1471-2334-14-1 24380631 PMC3880458

[B13] Lin Y. T. Wang Y. P. Wang F. D. Fung C. P. (2015). Community-Onset *Klebsiella Pneumoniae* Pneumonia in Taiwan: Clinical Features of the Disease and Associated Microbiological Characteristics of Isolates From Pneumonia and Nasopharynx. Front. Microbiol. 9. doi: 10.3389/fmicb.2015.00122 PMC4332370 25741336

[B14] Magiorakos A. P. Srinivasan A. Carey R. B. Carmeli Y. Falagas M. E. Giske C. G.. (2012). Multidrug-Resistant, Extensively Drug-Resistant and Pandrug-Resistant Bacteria: An International Expert Proposal for Interim Standard Definitions for Acquired Resistance. Clin. Microbiol. Infect. 18 (3), 268–281. doi: 10.1111/j.1469-0691.2011.03570.x 21793988

[B15] Metlay J. P. Waterer G. W. Long A. C. Anzueto A. Brozek J. Crothers K.. (2019). Diagnosis and Treatment of Adults With Community-Acquired Pneumonia. An Official Clinical Practice Guideline of the American Thoracic Society and Infectious Diseases Society of America. Am. J. Respir. Crit. Care Med. 200, e45–e67. doi: 10.1164/rccm.201908-1581ST 31573350 PMC6812437

[B16] Pan Y. J. Lin T. L. Lin Y. T. Su P. A. Chen C. T. Hsieh P. F.. (2015). Identification of Capsular Types in Carbapenem-Resistant *Klebsiella Pneumoniae* Strains by *Wzc* Sequencing and Implications for Capsule Depolymerase Treatment. Antimicrob. Agents Chemother. 59, 1038–1047. doi: 10.1128/AAC.03560-14 25451047 PMC4335867

[B17] Rodríguez-Baño J. Gutiérrez-Gutiérrez B. Machuca I. Pascual A. (2018). Treatment of Infections Caused by Extended-Spectrum-Beta-Lactamase-, AmpC-, and Carbapenemase-Producing *Enterobacteriaceae* . Clin. Microbiol. Rev. 31 (2), e00079–e00017. doi: 10.1128/CMR.00079-17 29444952 PMC5967687

[B18] Russo T. A. Marr C. M. (2019). Hypervirulent *Klebsiella Pneumoniae* . Clin. Microbiol. Rev. 32 (3), e00001–e00019. doi: 10.1128/CMR.00001-19 31092506 PMC6589860

[B19] Russo T. A. Olson R. Fang C. T. Stoesser N. Miller M. MacDonald U.. (2018). Identification of Biomarkers for Differentiation of Hypervirulent *Klebsiella Pneumoniae* From Classical *K. Pneumoniae* . J. Clin. Microbiol. 56 (9), e00776–18. doi: 10.1128/JCM.00776-18 PMC6113484 29925642

[B20] Shields R. K. Nguyen M. H. Chen L. Press E. G. Potoski B. A. Marini R. V.. (2017). Ceftazidime-Avibactam Is Superior to Other Treatment Regimens Against Carbapenem-Resistant Klebsiella Pneumoniae Bacteremia. Antimicrob. Agents Chemother. 61 (8), e00883–e00817. doi: 10.1128/AAC.00883-17 28559250 PMC5527595

[B21] Song J. H. Oh W. S. Kang C. I. Chung D. R. Peck K. R. Ko K. S.. (2008). Epidemiology and Clinical Outcomes of Community-Acquired Pneumonia in Adult Patients in Asian Countries: A Prospective Study by the Asian Network for Surveillance of Resistant Pathogens. Int. J. Antimicrob. Agents 31, 107–114. doi: 10.1016/j.ijantimicag.2007.09.014 18162378 PMC7134693

[B22] Tamma P. D. Aitken S. L. Bonomo R. A. Mathers A. J. van Duin D. Clancy C. J. (2021). Infectious Diseases Society of America Guidance on the Treatment of Extended-Spectrum β-Lactamase Producing *Enterobacterales* (ESBL-E), Carbapenem-Resistant *Enterobacterales* (CRE), and *Pseudomonas Aeruginosa* With Difficult-to-Treat Resistance (DTR-*P. Aeruginosa*). Clin. Infect. Dis. 72 (7), 1109–1116. doi: 10.1093/cid/ciab295 33830222

[B23] Vincent J. L. Moreno R. Takala J. Willatts S. De Mendonça A. Bruining H.. (1996). The SOFA (Sepsis-Related Organ Failure Assessment) Score to Describe Organ Dysfunction/Failure. On Behalf of the Working Group on Sepsis-Related Problems of the European Society of Intensive Care Medicine. Intensive Care Med. 22 (7), 707–710. doi: 10.1007/BF01709751 8844239

[B24] Yahav D. Giske C. G. Grāmatniece A. Abodakpi H. Tam V. H. Leibovici L. (2020). New β-Lactam-β-Lactamase Inhibitor Combinations. Clin. Microbiol. Rev. 34 (1), e00115–e00120. doi: 10.1128/CMR.00115-20 PMC7667665 33177185

[B25] Yang C. Y. Lee C. H. Hsieh C. C. Ko W. C. Lee C. C. (2018). Etiology of Community-Onset Monomicrobial Bacteremic Pneumonia and Its Clinical Presentation and Outcome: *Klebsiella* and *Pseudomonas* Matters. J. Infect. Chemother. 24 (1), 53–58. doi: 10.1016/j.jiac.2017.08.019 29017829

[B26] Zhu J. Wang T. Chen L. Du H. (2021). Virulence Factors in Hypervirulent *Klebsiella Pneumoniae* . Front. Microbiol. 12. doi: 10.3389/fmicb.2021.642484 PMC8060575 33897652

