# Supplementary material for: Clinical and Microbiological Characteristics of Bacteremic Pneumonia Caused by Klebsiella pneumoniae
Source: Front Cell Infect Microbiol. 2022 Jun 23;12:903682. doi: 10.3389/fcimb.2022.903682 (PMC9259976; doi:10.3389/fcimb.2022.903682)
Supplement: Supplementary file 1 [file Table_1.docx]

Supplementary Material

# Supplementary Tables

**Table S1. Characteristics of 28-day survivors and non-survivors of CAP**

| Variable | Survivors  n=33 (63.5%) | Non-survivors  n=19 (36.5%) | P value |
| --- | --- | --- | --- |
| **Demographics** |  |  |  |
| Age | 78 (66-86) | 84 (77-88) | 0.115 |
| Male sex | 29 (87.9) | 12 (63.2) | 0.074 |
| **Underlying disease** |  |  |  |
| Charlson Comorbidity Index | 8 (6-9) | 8 (7.5-9.5) | 0.218 |
| Diabetes mellitus | 14 (42.4) | 6 (31.6) | 0.558 |
| Congestive heart failure | 8 (24.2) | 2 (10.5) | 0.293 |
| Chronic kidney disease | 11 (33.3) | 6 (31.6) | 1.000 |
| Hemodialysis | 3 (9.1) | 1 (5.3) | 1.000 |
| Liver cirrhosis | 3 (9.1) | 1 (5.3) | 1.000 |
| Cerebral vascular disease | 9 (27.3) | 7 (36.8) | 0.541 |
| Malignancy | 12 (36.4) | 9 (47.4) | 0.559 |
| Immunosuppression | 8 (24.2) | 5 (26.3) | 1.000 |
| **Prior antibiotic exposure** |  |  |  |
| Any antibiotic | 6 (18.2) | 6 (31.6) | 0.317 |
| 1st / 2nd generation CEF | 3 (9.1) | 1 (5.3) | 1.000 |
| 3rd / 4th generation CEF | 3 (9.1) | 3 (15.8) | 0.656 |
| BLBLI | 4 (12.1) | 3 (15.8) | 0.697 |
| Carbapenem | 1 (3) | 0 (0) | 1.000 |
| Fluoroquinolone | 3 (9.1) | 2 (10.5) | 1.000 |
| Aminoglycoside | 0 (0) | 1 (5.3) | 0.365 |
| **Disease severity** |  |  |  |
| SOFA score | 8 (5-12) | 11 (9-13.5) | **0.028** |
| APACHE II score | 26 (21-33) | 35 (28.5-42) | **0.001** |
| Septic shock at diagnosis | 17 (51.5) | 18 (94.7) | **0.002** |
| **Microbiological** **characteristics** |  |  |  |
| Hvpervirulent KP | 23 (69.7) | 9 (47.4) | 0.144 |
| ESBL/AmpC-producing KP | 3 (9.1) | 1 (5.3) | 1.000 |
| Carbapenemase-producing KP | 0 (0) | 0 (0) | - |
| **Treatment** |  |  |  |
| Appropriate empirical therapy | 30 (90.9) | 18 (94.7) | 1.000 |
| Appropriate definite therapy | 33 (100) | 18 (94.7) | 0.365 |

Data are presented as median (interquartile range) for continuous variables, and number (percent) for categorical variables. Categorical variables were evaluated using the Fisher’s exact test. Continuous variables were evaluated using the Mann–Whitney U test. Statistically significant *P* values are highlighted in bold. CAP, community-acquired pneumonia; CEF, cephalosporin; BLBLI, β-lactam-β-lactamase inhibitor; KP, *Klebsiella pneumoniae*; ESBL, extended-spectrum beta-lactamase.

**Table S2. Logistic regressions for variables associated with 28-day mortality for CAP**

| Variable | Univariate | | Multivariate | |
| --- | --- | --- | --- | --- |
|  | *P* value | OR (95% CI) | *P* value | aOR (95% CI) |
| Male sex | **0.044** | 0.24 (0.06-0.96) | 0.168 | 0.31 (0.06-1.64) |
| SOFA score | **0.024** | 1.2 (1.02-1.4) | - | - |
| APACHE II score | **0.003** | 1.15 (1.05-1.26) | **0.037** | 1.1 (1.01-1.21) |
| Septic shock at diagnosis | **0.009** | 16.94 (2.02-142.03) | 0.064 | 8.25 (0.88-77.24) |

Statistically significant *P* values are highlighted in bold. CAP, community-acquired pneumonia; OR, odds ratio; CI confidence interval; aOR, adjusted odds ratio.

**Table S3. Characteristics of 28-day survivors and non-survivors of nosocomial pneumonia**

| Variable | Survivors  n=39 (39.8%) | Non-survivors  n=59 (60.2%) | P value |
| --- | --- | --- | --- |
| **Demographics** |  |  |  |
| Age | 69 (56.5-81) | 77 (61.5-86) | 0.087 |
| Male sex | 29 (74.4) | 44 (74.6) | 1.000 |
| LOS before pneumonia, days | 14 (8.5-36.5) | 24 (9.5-51) | 0.351 |
| VAP | 24 (61.5) | 26 (44.1) | 0.103 |
| **Underlying disease** |  |  |  |
| Charlson Comorbidity Index | 7 (5-9.5) | 9 (7-11) | **0.003** |
| Diabetes mellitus | 14 (35.9) | 21 (35.6) | 1.000 |
| Congestive heart failure | 6 (15.4) | 15 (25.4) | 0.316 |
| Chronic kidney disease | 9 (23.1) | 21 (35.6) | 0.263 |
| Hemodialysis | 6 (15.4) | 14 (23.7) | 0.443 |
| Liver cirrhosis | 2 (5.1) | 5 (8.5) | 0.699 |
| Cerebral vascular disease | 9 (23.1) | 15 (25.4) | 1.000 |
| Malignancy | 15 (38.5) | 32 (54.2) | 0.151 |
| Immunosuppression | 15 (38.5) | 32 (54.2) | 0.151 |
| **Prior antibiotic exposure** |  |  |  |
| Any antibiotic | 30 (76.9) | 45 (76.3) | 1.000 |
| 1st / 2nd generation CEF | 6 (15.4) | 3 (5.1) | 0.150 |
| 3rd / 4th generation CEF | 8 (20.5) | 26 (44.1) | **0.018** |
| BLBLI | 13 (33.3) | 28 (47.5) | 0.211 |
| Carbapenem | 14 (35.9) | 31 (52.5) | 0.147 |
| Fluoroquinolone | 14 (35.9) | 17 (28.8) | 0.510 |
| Aminoglycoside | 5 (12.8) | 4 (6.8) | 0.477 |
| **Disease severity** |  |  |  |
| SOFA score | 7 (5-9) | 12 (9-16) | **<0.001** |
| APACHE II score | 24 (19-29.5) | 33 (27-38) | **<0.001** |
| Septic shock at diagnosis | 17 (43.6) | 45 (76.3) | **0.001** |
| **Microbiological** **characteristics** |  |  |  |
| Hvpervirulent KP | 7 (17.9) | 9 (15.3) | 0.784 |
| ESBL/AmpC-producing KP | 7 (17.9) | 19 (32.2) | 0.161 |
| Carbapenemase-producing KP | 16 (41) | 31 (52.5) | 0.305 |
| **Treatment** |  |  |  |
| Appropriate empirical therapy | 31 (79.5) | 44 (74.6) | 0.633 |
| Appropriate definite therapy | 38 (97.4) | 46 (78) | **0.007** |

Data are presented as median (interquartile range) for continuous variables, and number (percent) for categorical variables. Categorical variables were evaluated using the Fisher’s exact test. Continuous variables were evaluated using the Mann–Whitney U test. Statistically significant *P* values are highlighted in bold. LOS, length of stay; VAP, ventilator-associated pneumonia; CEF, cephalosporin; BLBLI, β-lactam-β-lactamase inhibitor; KP, *Klebsiella pneumoniae*; ESBL, extended-spectrum beta-lactamase.

**Table S4. Logistic regressions for variables associated with 28-day mortality for nosocomial pneumonia**

| Variable | Univariate | | Multivariate | |
| --- | --- | --- | --- | --- |
|  | *P* value | OR (95% CI) | *P* value | aOR (95% CI) |
| Age | 0.072 | 1.02 (1-1.05) |  |  |
| Charlson Comorbidity Index | **0.003** | 1.24 (1.08-1.43) | **0.004** | 1.28 (1.08-1.51) |
| Previous exposure, 3^rd^/4^th^ CEF | **0.019** | 3.05 (1.2-7.75) |  |  |
| SOFA score | **<0.001** | 1.3 (1.15-1.47) | **<0.001** | 1.32 (1.15-1.52) |
| APACHE II score | **<0.001** | 1.11 (1.05-1.18) |  |  |
| Septic shock at diagnosis | **0.001** | 4.16 (1.74-9.95) |  |  |
| Appropriate definite therapy | **0.025** | 0.09 (0.01-0.74) | **0.046** | 0.1 (0.01-0.96) |

Statistically significant *P* values are highlighted in bold. OR, odds ratio; CI confidence interval; aOR, adjusted odds ratio; CEF, cephalosporin.
